# Nat-UV DB: A Natural Products Database Underlying of Veracruz-Mexico

**DOI:** 10.12688/f1000research.161261.1

**Published:** 2025-02-04

**Authors:** Edgar López-López, Ana Margarita Hernández-Segura, Carlos Lara-Cuellar, Carolina Barrientos-Salcedo, Carlos M. Cerda-García-Rojas, José L. Medina-Franco

**Affiliations:** 1 DIFACQUIM Research Group, Department of Pharmacy, School of Chemistry, Universidad Nacional Autonoma de Mexico, Mexico City, Mexico City, 04510, Mexico; 2 Department of Chemistry and Graduate Program in Pharmacology, Center for Research and Advanced Studies of the National Polytechnic Institute, Mexico City, Mexico City, 07000, Mexico; 3 Laboratorio de Química Médica y Quimiogenómica, Facultad de Bioanálisis Campus Veracruz, Universidad Veracruzana, Veracruz, Veracruz, 91700, Mexico

**Keywords:** Biodiversity; Chemical diversity; Chemoinformatics; Natural products; Chemical multiverse; Chemical space

## Abstract

**Background:**

Natural products databases are well-structured data sources that offer new molecular development opportunities in drug discovery, agrochemistry, food, cosmetics, and several other research disciplines or chemical industries. The crescent world’s interest in the development of these databases is related to the exploration of chemical diversity in geographical regions with rich biodiversity.

**Methods:**

In this work, we introduce and discuss Nat-UV DB, the first natural products database from a coastal zone of Mexico. We discuss its construction, curation, and chemoinformatic characterization of their content, and chemical space coverage compared with other compound databases, like approved drugs, and other Mexican (BIOFACQUIM and UNIIQUIM databases) and the Latin American natural products database (LaNAPDB).

**Results:**

Nat-UV DB comprises 227 compounds that contain 112 scaffolds, of which 52 are not present in previous natural product databases. The compounds present in Nat-UV DB have a similar size, flexibility, and polarity to previously reported natural products and approved drug datasets.

**Conclusions:**

Nat-UV DB compounds have a higher structural and scaffold diversity than the approved drugs, but they have low structural and scaffold diversity in contrast with other natural products in the reference datasets. This database serves as a valuable addition to the global natural products landscape, bridging gaps in exploring biodiversity-rich regions.

## Introduction

Mexico is one of the most biodiverse countries in the world, which has a large list of endemic organisms. ^1, 2^ At the same time, the state of Veracruz, Mexico, is a coastal region next to the Gulf of Mexico, which has a large diversity in its geographic landscapes and weather, conditions, which have contributed to the increase in biodiversity, and is considered one of the most biodiverse states in the country. ^3^ It has been reported that the state of Veracruz houses 34% of the total species in Mexico, which highlights the importance of the systematic study of their chemical diversity. ^4^

Natural products have demonstrated their key role in developing new drugs, materials, nutraceuticals, pesticides, and insecticides, which justify their study. ^5, 6^ Nowadays it is possible to establish structure-properties relationships using bioinformatics and chemoinformatics methodologies. ^7^ To achieve this goal, it is necessary to condense, organize, and curate the databases. Recent efforts in Latin America have been developed on the construction of natural products databases that have contributed to the understanding of Latin American traditional medicine, and to accelerate the rational use of natural products in this geographical region. ^8^ Particularly in Mexico, there are two compound databases ^9, 10^ that are mainly focused on the natural products identified in the central zone of Mexico. However, there are no reports of databases from the most biodiverse regions in this country.

**Figure 1.**
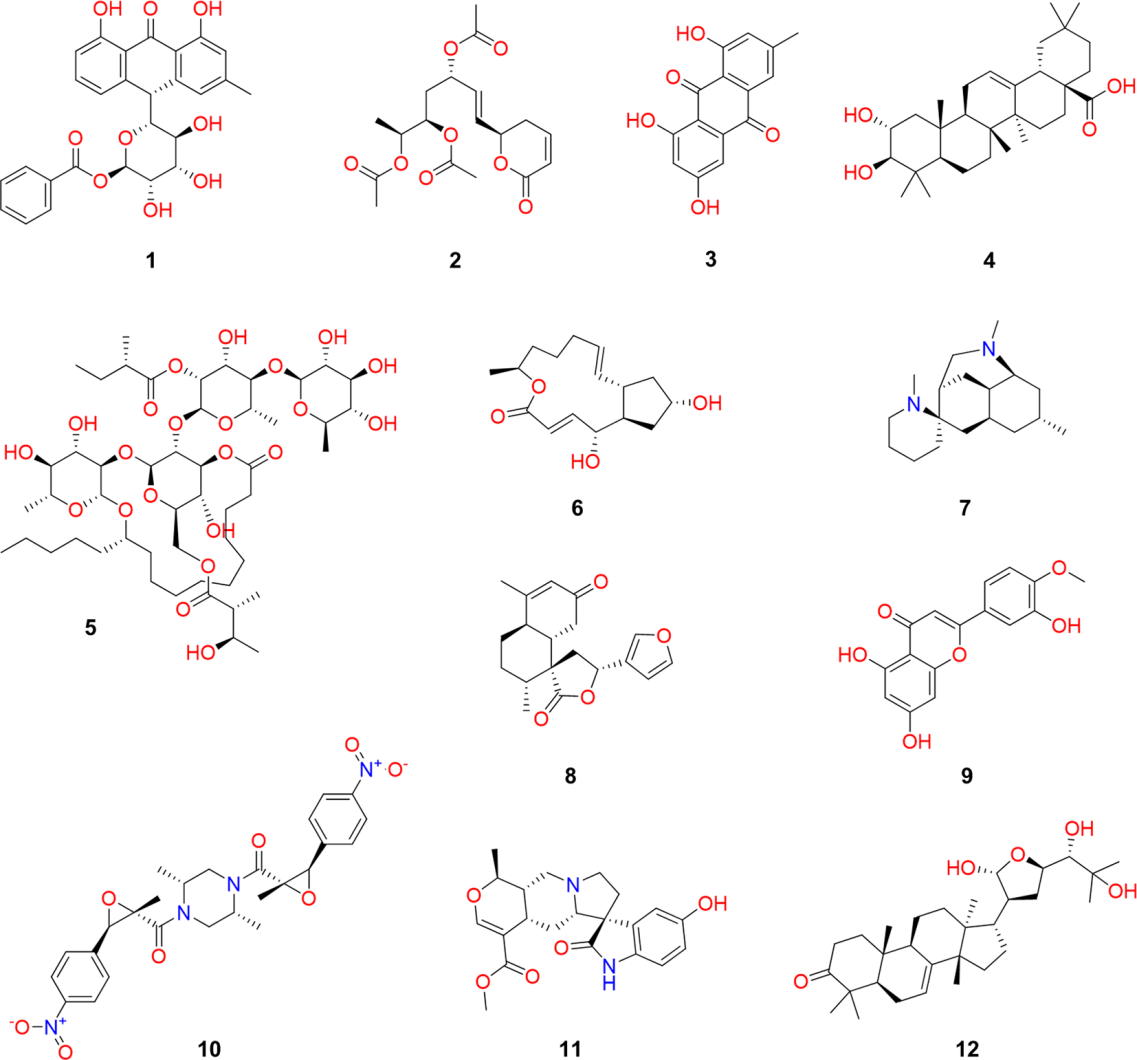
Representative natural products of the state of Veracruz, Mexico.

Figure 1 illustrates representative chemical structures that have been obtained from natural resources collected in the state of Veracruz, which are distinguished by their structural diversity, ^11– 21^ and their great applicability domain in medicine ( *e.g.* to develop antimicrobial and anticancer drugs), cosmetology ( *e.g.* to develop skincare molecules), nutrition ( *e.g.* to develop nutraceuticals), agriculture ( *e.g.* to develop biopesticides and insecticides), and many other applications. ^22– 25^

In the present scientific context, it is possible to establish highly efficient virtual screening protocols using chemoinformatics methods, natural product databases have covered a worldly interest in the past 20 years. ^26, 27^ Multiple commercial and open-access natural product databases are available as valuable resources for molecular design. It is expected that databases will continue to grow in number and type. For example, focusing their creation on the organization of data and information on natural products based on their reported biological activity, chemical characterization method, geolocation, natural source, commercial availability, etc. ^27^ Web applications like COCONUT 2.0 (the COlleCtion of Open NatUral producTs) is an an excellent resource freely available at https://coconut.naturalproducts.net/ to unify and standardize multiple natural product databases, ^28^ which facilitates the systematic filtering of multipurpose data useful for chemoinformatic and natural products research.

The main objective of this work is to introduce Nat-UV DB, a database of natural products isolated and characterized in the state of Veracruz, Mexico. We also discuss a systematic analysis using chemoinformatics methods, identifying endemic natural products, and studying their chemical diversity.

## Methods

### Database construction and curation

**Figure 2.**
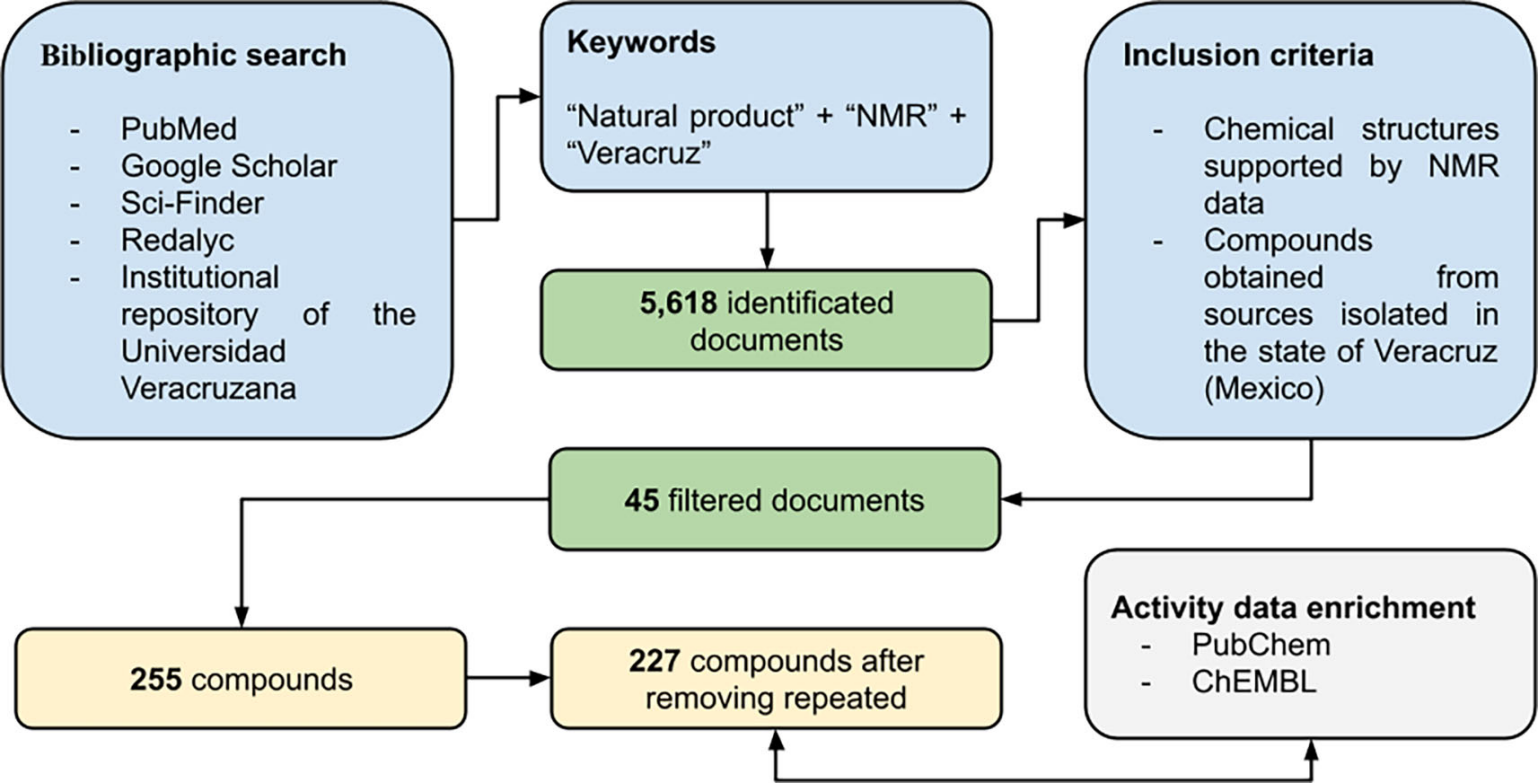
Workflow used to construct the Nat-UV database.

The database of natural products from the state of Veracruz was assembled from a literature search. For the construction of the first version of NAT-UV DB, PubMed, Google Scholar, Sci-Finder, Redalyc, and the institutional repository of the Universidad Veracruzana (Mexico) databases were searched using the keywords “natural product”, “NMR”, and “Veracruz.” We collected information from research articles, and bachelor, master, and doctorate theses from universities and research centers. To complement the data mining, two additional criteria were used for the final selection of the literature used to construct the database. The first filter was that the elucidation of the reported chemical structures has been supported by nuclear magnetic resonance (NMR). The second one was that the compounds identified were obtained from a natural source from any region in the state of Veracruz (Mexico). The search was generated by publication year from 1970 to June of 2024. We want to emphasize that this is the first version of Nat-UV DB; future versions will have natural products from more years, and more research repositories, to assemble a database representative of the entire biodiversity of the state of Veracruz. For each collected molecule, their isomeric SMILES strings ^29^ were generated with ChemBioDraw Ultra V.13, maintaining the stereochemistry reported in the primary literature. ^30^ With the module’Wash’, from the molecular operating environment (MOE) program, version 2024, ^31^ the database was curated, maintaining without changes the stereochemistry reported of each molecule. This was done to normalize and collect the most relevant information from the molecules. The data curation involved the elimination of salts, the adjustment of the protonation states, and the elimination of the duplicated molecules. The default settings of the ‘Wash’ module were used. The information collected for each identified compound is organized according to the natural origin of its place of collection, like kingdom, genera, species, and geographical collection. Finally, the list of curated compounds was manually cross-referenced to PubChem ^32^ and ChEMBL v.34 ^33^ databases, which enabled the annotation of databases with the bioactivities that have been associated with each chemical structure ( Figure 2). For those compounds reported in theses, and which were evaluated in a biological test, the biological activity was also included in the database.

### Reference data sets

In order to characterize the chemical diversity of Nat-UV DB and to explore its chemical space coverage, approved drugs ^34^ and the Latin American natural products compound database (LaNAPDB) ^35^ were used to compare their chemical structures and properties. The structure files used in this work were taken from open repositories of previously published analyses of natural products databases. ^36^ The structures of the reference compounds were curated using the same procedure described to prepare Nat-UV DB. Table 1 summarizes Nat-UV DB and the reference databases and the number of compounds. Of note, the reference collections included data sets of natural products, including two from Mexico.

**Table 1.** Reference databases compared with Nat-UV DB.

Database	Description	Size *	Reference
Approved drugs (DrugBank v. 2024.0)	Drugs approved for clinical use	2,144 ^⧫^	^34^
LANaPDB 2.0	Latinoamerican natural products database with chemicals from Brazil, Colombia, Costa Rica, El Salvador, Mexico, Panama, and Peru	13,579	^36^
BIOFACQUIM	Natural products from, Mexico	531	^37^
UNIIQUIM	Natural products from, Mexico	855	^10^
Nat-UV DB	Natural products from the state of Veracruz (Mexico)	227	-

* Number of compounds after data curation.

^⧫^ Small molecules.

### Druglikness profiling

The curated Nat-UV DB database was characterized by calculating six physicochemical properties of pharmaceutical interest, namely: molecular weight (MW), octanol/water partition coefficient (ClogP), topological surface area (TPSA), number of rotatable bonds (RB), number of H-bond donor atoms (HBD), and number of H-bond acceptor atoms (HBA). The statistical analysis was done with the program DataWarrior v.06, ^38^ by calculating the mean, median, and standard deviation of the calculated properties. Based on these statistics Nat-UV DB was further compared with other databases (LANAPDB, BIOFACQUIM, UNIIQUIM, and approved drugs from DrugBank) ( Table 1). The systematic comparison was generated using the Python programming language. The code is freely available at https://github.com/EdgL2/Nat-UV-DB.

### Scaffold content analysis of Nat-UV DB

The most frequent and unique molecular scaffolds of Nat-UV DB and reference databases ( Table 1) were computed using the scaffold definition of Bemis and Murcko. ^39^ This analysis was done using Python, the code of which is freely available at https://github.com/EdgL2/Nat-UV-DB.

### Visualization of the chemical space

In order to generate a visual representation of the chemical space of Nat-UV DB, the fingerprint ECFP4 (1024 bits) was calculated for each compound ^40^ and the visualization was done using t-distributed stochastic neighbor embedding (t-SNE). ^41^ The selection of this visualization method was based on recent studies that support its utility for the systematic study of small and large datasets in terms of neighborhood preservation and visualization capabilities. ^42^ The ECFP4 fingerprint and the t-SNE coordinates were calculated in KNIME software. The optimization parameters we used in t-SNE were dimensions (3), iterations (10,000), theta (0.3), perplexity (30.0), and number of threats (8), using 28 as the seed number. The interactive visualization was implemented using DataWarrior software, version 06. ^43^ The KNIME workflow and data generated are freely available in the Software availability section.

### Chemical diversity analysis

To compare the chemical diversity of Nat-UV DB with the reference data sets, we employed the consensus diversity (CD) plot which is a simple two-dimensional graph that helps to visualize and compare the diversity of several compound data sets considering multiple representations such as chemical scaffolds, and fingerprint-based diversity. ^44^ In this study, the CD plot was generated using the median paired similarity (ECFP4-1024 bits)/Tanimoto; x-axis) and the median paired scaffold similarity (Bemis-Murck representations using ECFP4-1014 bits/Tanimoto; y-axis). ^44^ Both are established and are representative metrics of the scaffold and fingerprint-based diversity. ^45^ Subsets of the compounds were retrieved from control data sets ( Table 1). The workflow implemented in KNIME software is available in the Software availability section.

## Results and discussion

In this section, we present the results of the construction of the Nat-UV database followed by a descriptive analysis of the contained data, and the chemoinformatic characterization in terms of physicochemical properties, scaffold content, chemical space coverage, and consensus chemical diversity.

### Nat-UV database

As described in the Methods section, the scientific papers and thesis that complied with the inclusion criteria were selected. Each of the 45 scientific documents selected (1 Doctorate thesis degree; 8 Master thesis degrees; 36 research articles) was analyzed individually to extract manually the chemical structures of each identified natural product. The Nat-UV DB contains information that allows identifying the bibliography precedence of the data. For example: compound name, reference, digital object identifier (DOI), and publication year. Also, it contains data related to the natural source precedence of the data. For example: kingdom, genus, species, and geographical location of the collection of the natural source. Additionally, we added cross-referenced IDs with other databases (e.g. PubChem and ChEMBL). Finally, we manually cross-referenced each compound with their reported bioactivity contained in ChEMBL v. 34.

**Figure 3.**
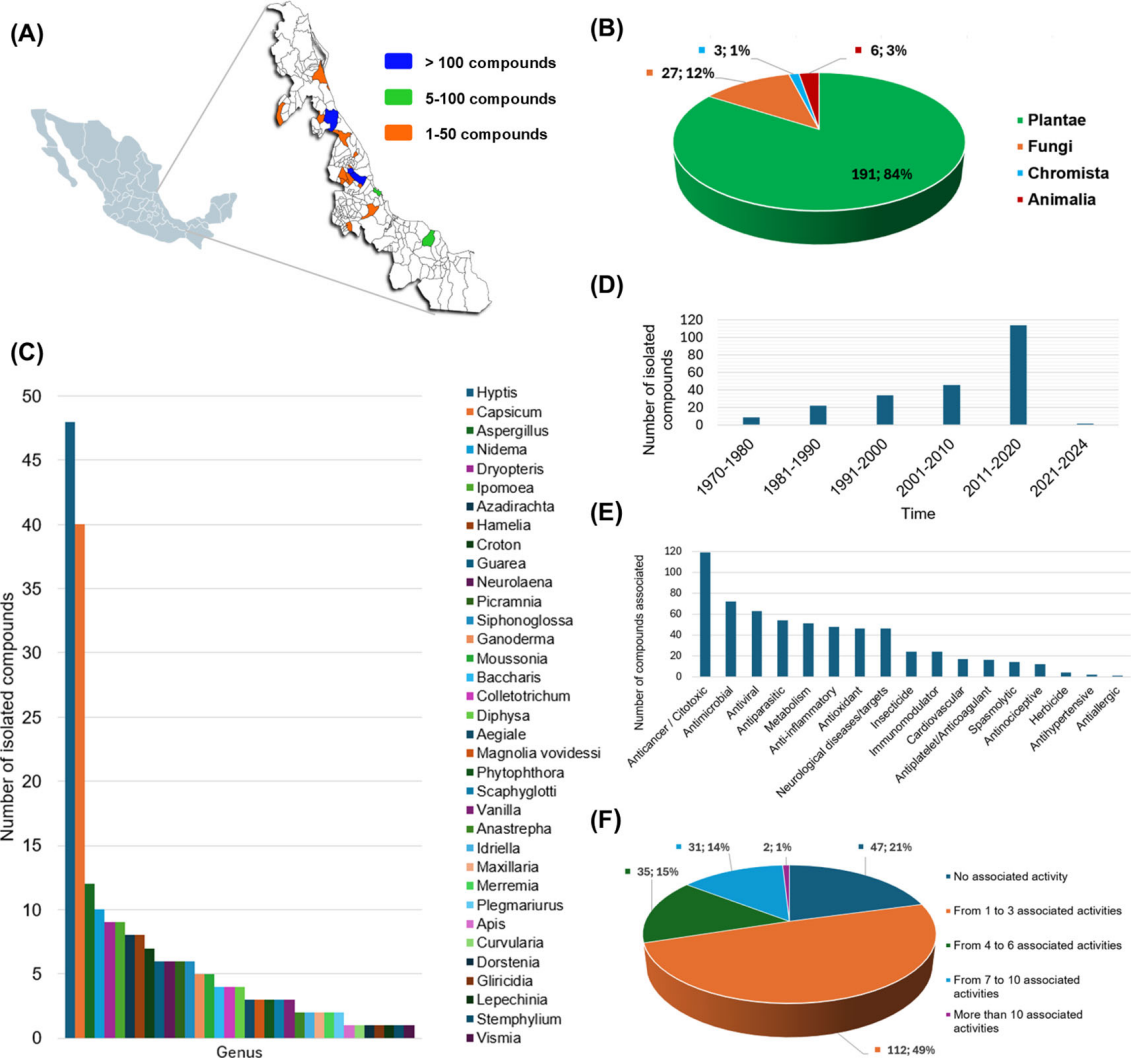
Descriptive analysis of the Nat-UV DB. (A) Geographical collection of natural resources studied in this work, and the number of compounds obtained by each region; (B) Quantification of compounds contained in this database by genus; (C) Quantification of compounds contained in this database by specie precedence; (D) Number of isolated compounds by decades; (E) Associated bioactivity for the compounds contained in this database; and (F) Multi-activity landscape of compounds contained in this database.

The current version of Nat-UV DB has 227 compounds collected from different geographical zones of Veracruz ( Figure 3A), mainly isolated by different kinds of gender plants ( Figure 3B). For example, the gender *Hyptis*, *Capsicum*, *Nidema*, *Dryopteris*, *Ipomoea*, *Azadirachta*, *Hamelia*, *Croton*, and *Guarea* are examples of the most frequently studied. Other species from other kingdoms also stand out as *Aspergillus*, *Ganoderma*, Colletotrichum, and *Aegiale* ( Figure 3C). Figure 1D illustrates the distribution of compounds per year reported since 1970 to date. Finally, 79% of the compounds contained in this database have been associated with almost one bioactivity report ( Figure 2E, D) which highlights compounds with anticancer and antimicrobial (antibacterial or antifungal) activities.

### Molecular scaffolds

**Figure 4.**
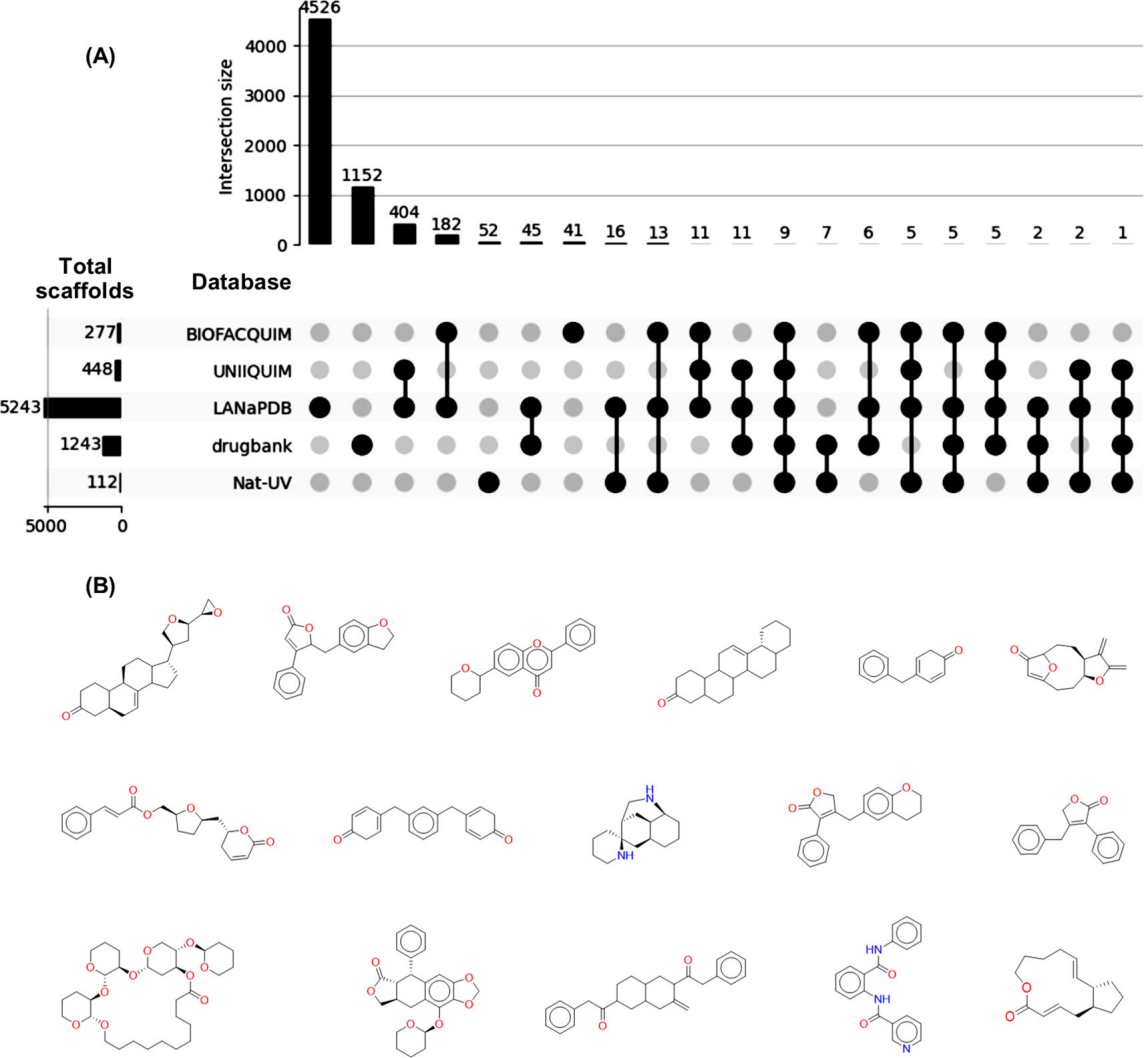
Unique scaffold content in Nat-UV DB. (A) Shared scaffolds of Nat-UV DB and reference datasets for natural products (LANaPDB, BIOFACQUIM, and UNIIQUIM) and approved drugs (Drugbank); (B) Representative unique scaffolds contained in Nat-UV DB.

From the total number of compounds contained in Nat-UV DB (227 compounds), 112 scaffolds were identified, of which 52 (52/112; 46%) are unique. Namely, Nat-UV DB contains scaffolds (52) that have not been reported previously in other Latin American datasets, and that are not present in the scaffolds collection of approved drugs ( Figure 4A). The most representative unique scaffolds of Nat-UV DB are shown in Figure 4B, highlighting the presence of derivatives of limonoids, butyrolactones, flavones, pentacyclic triterpenes, etc. The full list of unique scaffolds is available in the Data availability section.

There are previous reports of two Mexican natural products databases ( Table 1), but the BIOFACQUIM database is the unique one associated with collected geographical data. Interestingly, 74 compounds contained in this dataset were collected in the state of Veracruz (Mexico). This explains that 32 (32/112; 28%) scaffolds are shared in both databases ( Figure 4A). Also, 17 (17/112; 15%) scaffolds are shared between Nat-UV DB and UNIIQUIM, while 53 (53/112; 47%) scaffolds are shared between Nat-UV DB and the LaNaPDB. Finally, 24 (24/112; 21%) scaffolds were shared between Nat-UV DB and the approved drugs collection. In other words, Nat-UV DB contains some natural product scaffolds (60/112; 54%) that have been identified previously in Mexico and other Latin American countries or have been used as a drug.

### Molecular properties

**Figure 5.**
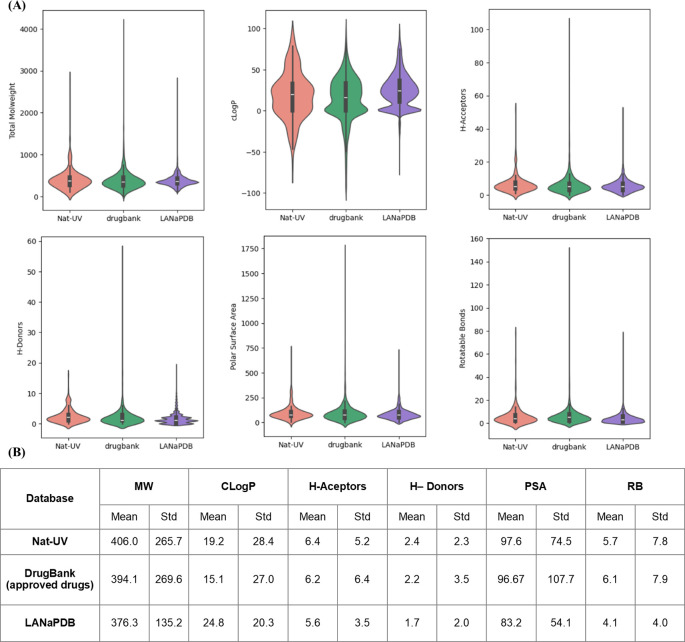
Violin plots for the drug-likeness physicochemical properties of Nat-UV DB and reference data sets. (A) The boxes inside of violins enclose data with values within the first and third quartile; (B) Summary statistics are included below each l plot. MW: molecular weight; C *log*P: octanol/water partition coefficient; H-bond acceptors: number of H-bond acceptor atoms; H-Donors: number of H-bond donor atoms; PSA: polar surface area; RB: number of rotatable bonds.

Figure 5 shows a violin plot of the distribution of the six drug-likeness properties calculated for Nat-UV DB. The distribution of the same properties for the two references used in this work was included in comparing the violin plots. ( Table 1). Intrinsic molecular properties like size, flexibility, and polarity are described by explicit molecular properties like weight (MW), coefficient of octanol/water partition (C *Log*P), number of H-acceptor and H-donors bonds, polar surface area (PSA), and number of rotatable bonds (RB) ( Figure 5A). Summary statistics are presented at the bottom of the violin plots ( Figure 5B).

According to Figure 5 the size (MW, HA, and HB), flexibility (RB), and permeability (PSA) profiling of Nat-UV are comparable with the control datasets. However, the polarity (C *Log*P) of the compounds contained in Nat-UV DB, LANaPDB, BIOFACQUIM, and UNIIQUIM is higher than the approved drugs, however, the Nat-UV DB exhibited a shorter distribution than each natural products databases. This finding agrees with previous reports indicating that natural products are slightly more hydrophobic than drugs approved for clinical use. ^36^

### Chemical space and diversity analysis

**Figure 6.**
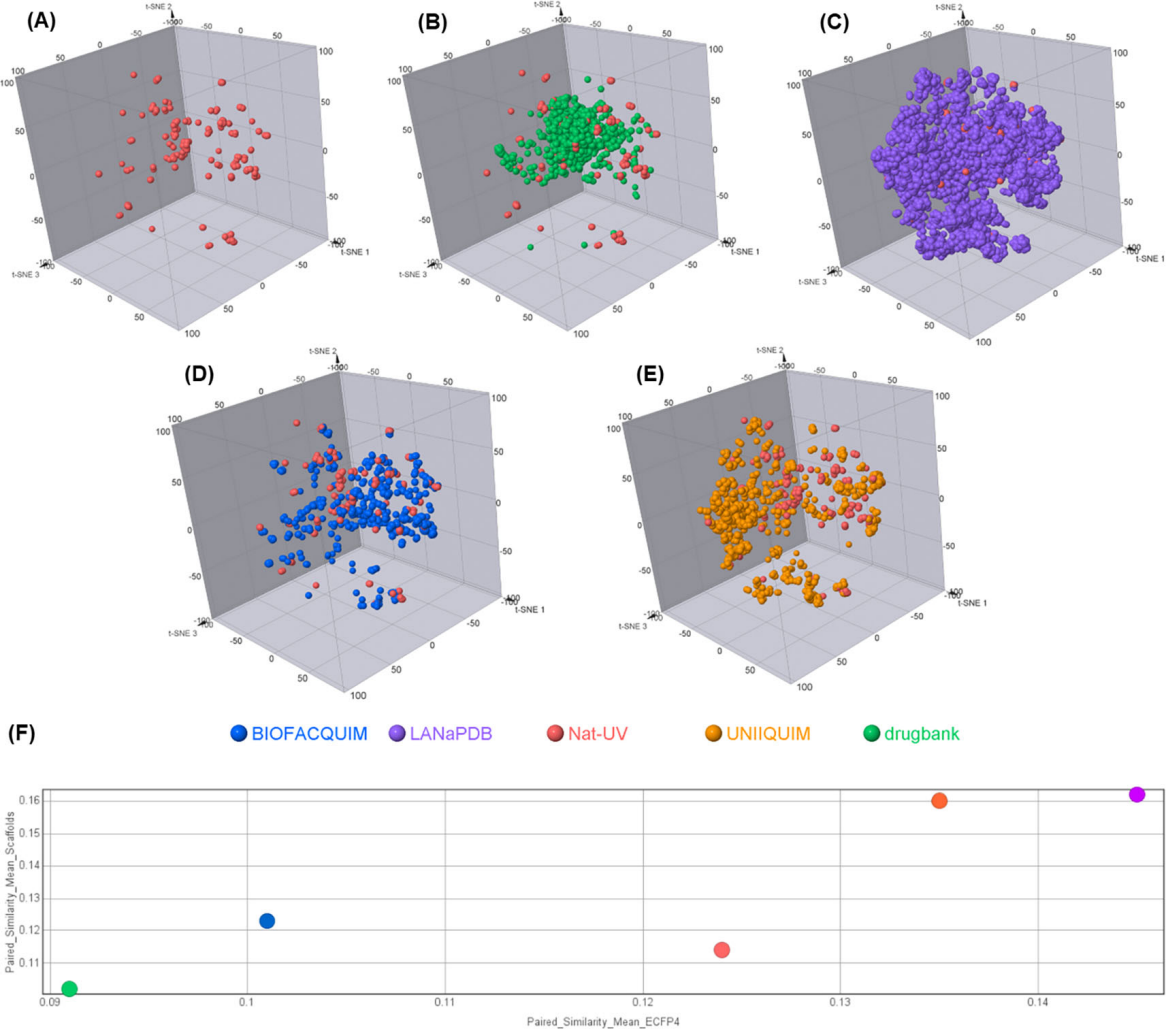
Visual representation of the chemical space coverage of Nat-UV DB and reference datasets based on ECFP4 and t-SNE as a visualization method. (A) Nat-UV DB; (B) Nat-UV DB and Drugbank (approved drugs); (C) Nat-UV DB and LANaPDB; (D) Nat-UV DB and BIOFACQUIM; (E) Nat-UV DB and UNIIQUIM; and (F) Consensus diversity plot of Nat-UV DB and the four reference datasets.

Figure 6 shows a visual representation of the chemical space of Nat-UV DB based on ECFP4 fingerprint using t-SNE. Figure 6(B-E) compares Nat-UV DB with other natural products databases (i.e. LANaPDB, BIOFACQUIM, and UNIIQUIM) and approved drugs. Interestingly, Nat-UV DB shares part of its chemical space with the approved drugs dataset, but Nat-UV DB compounds are more distributed in the three dimensions of the plot, which suggests that have a higher structural diversity than the approved drug dataset. However, LANaPDB (the largest dataset analyzed in this study) has an apparently higher structural diversity than the other studied datasets. To quantify the diversity of each dataset, the calculation of structural diversity and scaffold diversity were done ( Figure 6F). To quantify the diversity of each dataset, we calculated the mean of the paired similarity of the structures (x-axis) and scaffolds (y-axis) based on the similarity of each pair of compounds in the dataset using ECFP4 fingerprint and Tanimoto coefficient, where the higher values confirm a higher structural or scaffold diversity of the dataset. These results showed that Nat-UV DB has a higher structural and scaffold diversity than the approved drugs. However, it has low structural and scaffold diversity in contrast with UNIIQUIM and LANaPDB. Finally, Nat-UV DB shows a higher structural diversity than BIOFACQUIM, but a lower scaffold diversity than this one.

## Conclusions

Nat-UV DB is a compound database of natural products from the state of Veracruz in Mexico, which is a coastal zone reported with a large biodiversity. The open-access database contains 227 compounds reported from 1970 to June 2024, which is available at https://github.com/EdgL2/Nat-UV-DB. The compound database contains information of bibliographic resources for each compound, information about the collected species that come from, and cross-referenced bioactivity data. The chemoinformatic characterization and analysis of the coverage and diversity of Nat-UV DB in the chemical space suggest broad coverage, overlapping with regions in the approved drugs chemical space. The analysis also indicated that there are unique compounds in Nat-UV DB concerning other Mexican and Latin American natural products databases. The main perspectives of this work are to use Nat-UV DB to identify active compounds using virtual screening methods and continue to augment the size of Nat-UV DB from the new natural products that would be identified in the state of Veracruz, Mexico.

## Ethics and consent

Ethical approval and consent were not required.

## Data Availability

Zenodo: Nat-UV DB Data Availability. The datasets used in this work. https://doi.org/10.5281/zenodo.14715820. ^46^ This project contains the following underlying data:
•
FinalDB_ForPaper_DB_cured.csv: The Nat-UV compounds and approved drugs datasets. •
Most_Frecuent_Scaffolds_NatUVDB.xlsx: The most frequent scaffolds contained in Nat-UV DB. Data are available under the terms of the Creative Commons Attribution 4.0 International license (CC-BY 4.0).

## Software availability

Source code available from: https://github.com/EdgL2/Nat-UV-DB. ^47^

Archived software available from: https://doi.org/10.5281/zenodo.14715820. ^47^

The codes and workflow are available under the terms of the Creative Commons Attribution 4.0 International license (CC-BY 4.0).

## References

[1] Dávila-Aranda P Lira-Saade R Valdés-Reyna J : Endemic species of grasses in Mexico: a phytogeographic approach. *Biodivers. Conserv.* 2004;13:1101–1121. 10.1023/B:BIOC.0000018147.54695.b3

[2] Mapes C Basurto F : Biodiversity and edible plants of Mexico. Lira R Casas A Blancas J , editors. *Ethnobotany of Mexico. Ethnobiology.* New York, NY: Springer;2016. 10.1007/978-1-4614-6669-7_5

[3] Peterson AT Egbert SL Sánchez-Cordero V : Geographic analysis of conservation priority: endemic birds and mammals in Veracruz, Mexico. *Biol. Conserv.* 2000;93:85–94. 10.1016/S0006-3207(99)00074-9

[4] SEMARNAT: Informe de la situación del medio ambiente en México. 2015. Accessed 15 November 2024. Reference Source

[5] Chopra B Dhingra AK : Natural products: A lead for drug discovery and development. *Phytother. Res.* 2021;35:4660–4702. 10.1002/ptr.7099 33847440

[6] Zhang X Jiang M Niu N : Natural-product-derived carbon dots: From natural products to functional materials. *ChemSusChem.* 2017;11:11–24. 10.1002/cssc.201701847 29072348

[7] López-López E Medina-Franco JL : Toward structure-multiple activity relationships (SMARts) using computational approaches: A polypharmacological perspective. *Drug Discov. Today.* 2024;29:104046. 10.1016/j.drudis.2024.104046 38810721

[8] Gómez-García A Medina-Franco JL : Progress and impact of Latin American natural product databases. *Biomolecules.* 2022;12:1202. 10.3390/biom12091202 36139041 PMC9496143

[9] Pilón-Jiménez B Saldívar-González F Díaz-Eufracio B : BIOFACQUIM: A Mexican compound database of natural products. *Biomolecules.* 2019;9:31. 10.3390/biom9010031 30658522 PMC6358837

[10] UNIIQUIM: Lista de compuestos. 2024. Accessed 15 November 2024. Reference Source

[11] Hernandez-Medel MDR Garcia-Salmones I Santillan R : An anthrone from Picramnia antidesma. *Phytochemistry.* 1998;49:2599–2601. 10.1016/S0031-9422(98)00383-5

[12] Martínez-Fructuoso L Pereda-Miranda R Rosas-Ramírez D : Structure elucidation, conformation, and configuration of cytotoxic 6-heptyl-5,6-dihydro-2H-pyran-2-ones from hyptis species and their molecular docking to α-Tubulin. *J. Nat. Prod.* 2019;82:520–531. 10.1021/acs.jnatprod.8b00908 30601004

[13] Mendoza Cervantes G : Obtención de macrosporina a partir de Stemphylium lycopersici hongo fitopatógeno de papaya. 2006. Accessed 15 November 2024. Reference Source

[14] Gutiérrez-Rebolledo GA Garduño-Siciliano L García-Rodríguez RV : Anti-inflammatory and toxicological evaluation of Moussonia deppeana (Schldl. & Cham) hanst and verbascoside as a main active metabolite. *J. Ethnopharmacol.* 2016;187:269–280. 10.1016/j.jep.2016.04.033 27125592

[15] Hernández-Carlos B Bye R Pereda-Miranda R : Orizabins V−VIII, tetrasaccharide glycolipids from the Mexican Scammony Root (Ipomoea orizabensis). *J. Nat. Prod.* 1999;62:1096–1100. 10.1021/np9900627 10479311

[16] Espinoza C Couttolenc A Fernández JJ : Brefeldin-A: an antiproliferative metabolite of the fungus Curvularia trifolii collected from the Veracruz coral reef system, Mexico. *J. Mex. Chem. Soc.* 2016;60:79–82. Accessed 15 November 2024. Reference Source

[17] Cruz-Miranda OL Folch-Mallol J Martínez-Morales F : Identification of a huperzine A-producing endophytic fungus from Phlegmariurus taxifolius. *Mol. Biol. Rep.* 2019;47:489–495. 10.1007/s11033-019-05155-1 31659691

[18] García A Ramírez-Apan T Cogordan JA : Absolute configuration assignments by experimental and theoretical approaches of ent-labdane- and cis-ent-clerodane-type diterpenes isolated from Croton glabellus. *Can. J. Chem.* 2006;84:1593–1602. 10.1139/v06-164

[19] Rivera-Chávez J Coporo-Blancas D Morales-Jiménez J : One-step partial synthesis of (±)-asperteretone B and related hPTP1B1–400 inhibitors from butyrolactone I. *Bioorg. Med. Chem.* 2020;28:115817. 10.1016/j.bmc.2020.115817 33120077

[20] Paniagua-Vega D Cerda-García-Rojas CM Ponce-Noyola T : A new monoterpenoid oxindole alkaloid from Hamelia Patens micropropagated plantlets. *Nat. Prod. Commun.* 2012;7:1934578X1200701. 10.1177/1934578X1200701109 23285803

[21] Jimenez A Villarreal C Toscano RA : Limonoids from Swietenia humilis and Guarea grandiflora (Meliaceae). *Phytochemistry.* 1998;49:1981–1988. 10.1016/S0031-9422(98)00364-1

[22] Kaur K Jain M Kaur T : Antimalarials from nature. *Bioorg. Med. Chem.* 2009;17:3229–3256. 10.1016/j.bmc.2009.02.050 19299148

[23] Pereda-Miranda R Hernández L Villavicencio MJ : Structure and stereochemistry of pectinolides A-C, novel antimicrobial and cytotoxic 5,6-dihydro-α-pyrones from Hyptis pectinata. *J. Nat. Prod.* 1993;56:583–593. 10.1021/np50094a019 8496706

[24] Liu S Luo XH Liu YF : Emodin exhibits anti-acne potential by inhibiting cell growth, lipogenesis, and inflammation in human SZ95 sebocytes. *Sci. Rep.* 2023;13:21576. 10.1038/s41598-023-48709-x 38062074 PMC10703917

[25] Pastor R Bouzas C Tur JA : Beneficial effects of dietary supplementation with olive oil, oleic acid, or hydroxytyrosol in metabolic syndrome: Systematic review and meta-analysis. *Free Radic. Biol. Med.* 2021;172:372–385. 10.1016/j.freeradbiomed.2021.06.017 34153478

[26] Bajorath J Chávez-Hernández AL Duran-Frigola M : Chemoinformatics and artificial intelligence colloquium: progress and challenges in developing bioactive compounds. *J Cheminform.* 2022;14:82. 10.1186/s13321-022-00661-0 36461094 PMC9716667

[27] Sorokina M Steinbeck C : Review on natural products databases: where to find data in 2020. *J. Cheminform.* 2020;12:20. 10.1186/s13321-020-00424-9 33431011 PMC7118820

[28] Nainala VC Rajan K Kanakam SRS : COCONUT 2.0: A comprehensive overhaul and curation of the collection of open natural products database. *ChemRxiv.* 2024. 10.26434/chemrxiv-2024-fxq2s PMC11701633 39588778

[29] Weininger D : SMILES, a chemical language and information system. 1. Introduction to methodology and encoding rules. *J. Chem. Inf. Model.* 1988;28:31–36. 10.1021/ci00057a005

[30] Narayanaswamy VK Rissdörfer M Odhav B : Review on cambridgesoft ChemBioDraw ultra 13.0 v. *Int. J. Theor. Appl. Sci.* 2013;5:45–49.

[31] Molecular Operating Environment (MOE): Chemical computing group ULC, 910-1010 Sherbrooke St. W., Montreal, QC H3A 2R7, 2025. 2024.

[32] Kim S Chen J Cheng T : PubChem 2023 update. *Nucleic Acids Res.* 2023;51:D1373–D1380. 10.1093/nar/gkac956 36305812 PMC9825602

[33] Zdrazil B Felix E Hunter F : The ChEMBL database in 2023: a drug discovery platform spanning multiple bioactivity data types and time periods. *Nucleic Acids Res.* 2024;52:D1180–D1192. 10.1093/nar/gkad1004 37933841 PMC10767899

[34] Knox C Wilson M Klinger CM : DrugBank 6.0: the drugBank knowledgebase for 2024. *Nucleic Acids Res.* 2024;52:D1265–D1275. 10.1093/nar/gkad976 37953279 PMC10767804

[35] Gómez-García A Acuña Jiménez DA Zamora WJ : Navigating the chemical space and chemical multiverse of a unified Latin American natural product database: LANaPDB. *Pharmaceuticals.* 2023;16:1388. 10.3390/ph16101388 37895859 PMC10609821

[36] Gómez-García A Acuña Jiménez DA Zamora WJ : Latin American Natural Product Database (LANaPDB): An Update. *J. Chem. Inf. Model.* 2024;64:8495–8509. In press. 10.3390/ph16101388 39503579 PMC11600509

[37] Sánchez-Cruz N Pilón-Jiménez BA Medina-Franco JL : Functional group and diversity analysis of BIOFACQUIM: A Mexican natural product database. *F1000Res.* 2020;8(Chem Inf Sci):2071. 10.12688/f1000research.21540.2 32047598 PMC6993822

[38] Sander T Freyss J Korff M von : DataWarrior: An open-source program for chemistry aware data visualization and analysis. *J. Chem. Inf. Model.* 2015;55:460–473. 10.1021/ci500588j 25558886

[39] Bemis GW Murcko MA : The properties of known drugs. 1. Molecular frameworks. *J. Med. Chem.* 1996;39:2887–2893. 10.1021/jm9602928 8709122

[40] Rogers D Hahn M : Extended-connectivity fingerprints. *J. Chem. Inf. Model.* 2010;50:742–754. 10.1021/ci100050t 20426451

[41] Medina-Franco JL Sánchez-Cruz N López-López E : Progress on open chemoinformatic tools for expanding and exploring the chemical space. *J. Comput. Aided Mol. Des.* 2021;36:341–354. 10.1007/s10822-021-00399-1 34143323 PMC8211976

[42] Orlov AA Akhmetshin TN Horvath D : From high dimensions to human insight: Exploring dimensionality reduction for chemical space visualization. *Mol. Inform.* 2024;44:e202400265. 10.1002/minf.202400265 39633514 PMC11733715

[43] López-López E Naveja JJ Medina-Franco JL : DataWarrior: an evaluation of the open-source drug discovery tool. *Expert Opin. Drug Discov.* 2019;14:335–341. 10.1080/17460441.2019.1581170 30806519

[44] González-Medina M Prieto-Martínez FD Owen JR : Consensus diversity plots: a global diversity analysis of chemical libraries. *J. Cheminf.* 2016;8:63. 10.1186/s13321-016-0176-9 27895718 PMC5105260

[45] Dunn TB López-López E Kim TD : Exploring activity landscapes with extended similarity: is Tanimoto enough? *Mol. Inf.* 2023;42:e2300056. 10.1002/minf.202300056 37202375

[46] Medina-Franco JL : Zenodo: Nat-UV DB Data Availability. The datasets used in this work. 2025. 10.5281/zenodo.14715820

[47] Medina-Franco JL : Source code. *Archived software.* 2025. 10.5281/zenodo.14715820 Reference Source

