# Supplementary material for: Nat-UV DB: A Natural Products Database Underlying of Veracruz-Mexico
Source: F1000Res. 2025 Apr 24;14:Chem Inf Sci-157. Originally published 2025 Feb 4. [Version 2] doi: 10.12688/f1000research.161261.2 (PMC11966095; doi:10.12688/f1000research.161261.2)
Supplement: Peer review history

# Reviewer report: Reviewer response for version 1

**DOI:** 10.5256/f1000research.177266.r370989

**Published:** 2025-04-12

**Authors:** Kathia Maria Honorio

**Affiliations:** 1 Universidade de Sao Paulo, São Paulo, State of São Paulo, Brazil

The manuscript deals with a topic of great relevance in the areas of chemoinformatics, natural products and drug discovery. The approach used is appropriate and has led to the development of a database of compounds that will be extremely useful to the scientific community. I suggest minor revisions to the text:

 1) "...calculating six physicochemical properties of pharmaceutical interest..." - insert which tool was used to calculate the properties.

 2) "Figure 1D illustrates the distribution of compounds per year reported since 1970 to date." - would the correct term be Figure 3D?

 3) "Finally, 79% of the compounds contained in this database have been associated with almost one bioactivity report (Figure 2E, D)..." - would the correct term be "Figures 3E and F?

Is the work clearly and accurately presented and does it cite the current literature?

Yes

If applicable, is the statistical analysis and its interpretation appropriate?

Yes

Are all the source data underlying the results available to ensure full reproducibility?

Yes

Is the study design appropriate and is the work technically sound?

Yes

Are the conclusions drawn adequately supported by the results?

Yes

Are sufficient details of methods and analysis provided to allow replication by others?

Yes

Reviewer Expertise:

Computational medicinal chemistry

I confirm that I have read this submission and believe that I have an appropriate level of expertise to confirm that it is of an acceptable scientific standard.

# Response

**Published:** 2025-04-16

**Authors:** José L. Medina-Franco

**Affiliations:** Universidad Nacional Autónoma de México, Mexico

Dear Prof. Kathia Maria Honorio, We appreciate your valuable comments and suggestions. The second version of this revised work addresses all of them.

# Reviewer report: Reviewer response for version 1

**DOI:** 10.5256/f1000research.177266.r370987

**Published:** 2025-04-02

**Authors:** David J Newman

**Affiliations:** 1 NIH Special Volunteer, Wayne, USA

This is a well-described analysis of the chemical structures found in reports from the most-biodiverse state in Mexico. The authors have provided links to their sources, their analytical tools and the manner in which they have integrated their data sets to provide the first NP-structure analyses from this highly biodiverse area of Mexico.

 The authors have successfully linked analytical tools (in particular high-field NMR to confirm structures and biological sources have been identified.

 The various data sets provided (together with the methodologies used) will not only act as a building block for subsequent work but more importantly, will permit comparison(s) with both existing agents from other disparate areas not only in Mexico but also in other biodiverse geographic areas in Central and South America, in particular those from Brazil’s Atlantic Forest and various parts of Amazonia where there are databases extant.

Is the work clearly and accurately presented and does it cite the current literature?

Yes

If applicable, is the statistical analysis and its interpretation appropriate?

Yes

Are all the source data underlying the results available to ensure full reproducibility?

Yes

Is the study design appropriate and is the work technically sound?

Yes

Are the conclusions drawn adequately supported by the results?

Yes

Are sufficient details of methods and analysis provided to allow replication by others?

Yes

Reviewer Expertise:

NA

I confirm that I have read this submission and believe that I have an appropriate level of expertise to confirm that it is of an acceptable scientific standard.

# Reviewer report: Reviewer response for version 1

**DOI:** 10.5256/f1000research.177266.r370994

**Published:** 2025-03-20

**Authors:** Virginia Flores-Morales

**Affiliations:** 1 The Autonomous University of Zacatecas, Zacatecas, Mexico

The article is interesting and shows in a way very appropriate to the relevance of the results. The structure of the article allows an agile and pleasant reading. Future updates will make this database a reference for study in other regions of great diversity in Mexico. The findings are important and are very well reflected in the work.

 I would recommend to standardize PSA and change the TPSA of page 5. A subtlety. I also suggest in Figure 6, arrange the descriptors in colour by order of apparition, including panel F, to make the explanation more harmonious

Is the work clearly and accurately presented and does it cite the current literature?

Yes

If applicable, is the statistical analysis and its interpretation appropriate?

Yes

Are all the source data underlying the results available to ensure full reproducibility?

Yes

Is the study design appropriate and is the work technically sound?

Yes

Are the conclusions drawn adequately supported by the results?

Yes

Are sufficient details of methods and analysis provided to allow replication by others?

Yes

Reviewer Expertise:

My research lines focus on generating knowledge in the medicinal chemical study of synthetic molecules and natural products and Theoretical chemistry.

I confirm that I have read this submission and believe that I have an appropriate level of expertise to confirm that it is of an acceptable scientific standard.
